# Probabilistic prediction and ranking of human protein-protein interactions

**DOI:** 10.1186/1471-2105-8-239

**Published:** 2007-07-05

**Authors:** Michelle S Scott, Geoffrey J Barton

**Affiliations:** 1 School of Life Sciences Research, College of Life Sciences, University of Dundee, Scotland, UK

## Abstract

**Background:**

Although the prediction of protein-protein interactions has been extensively investigated for yeast, few such datasets exist for the far larger proteome in human. Furthermore, it has recently been estimated that the overall average false positive rate of available computational and high-throughput experimental interaction datasets is as high as 90%.

**Results:**

The prediction of human protein-protein interactions was investigated by combining orthogonal protein features within a probabilistic framework. The features include co-expression, orthology to known interacting proteins and the full-Bayesian combination of subcellular localization, co-occurrence of domains and post-translational modifications. A novel scoring function for local network topology was also investigated. This topology feature greatly enhanced the predictions and together with the full-Bayes combined features, made the largest contribution to the predictions. Using a conservative threshold, our most accurate predictor identifies 37606 human interactions, 32892 (80%) of which are not present in other publicly available large human interaction datasets, thus substantially increasing the coverage of the human interaction map. A subset of the 32892 novel predicted interactions have been independently validated. Comparison of the prediction dataset to other available human interaction datasets estimates the false positive rate of the new method to be below 80% which is competitive with other methods. Since the new method scores and ranks all human protein pairs, smaller subsets of higher quality can be generated thus leading to even lower false positive prediction rates.

**Conclusion:**

The set of interactions predicted in this work increases the coverage of the human interaction map and will help determine the highest confidence human interactions.

## Background

Protein-protein interactions perform and regulate fundamental cellular processes. The comprehensive study of such interactions on a genome-wide scale will lead to a clearer understanding of diverse cellular processes and of the molecular mechanisms of disease. Although the determination of interactions by small-scale laboratory techniques is impractical for a complete proteome on the grounds of cost and time, several experimental techniques now exist to determine protein-protein interactions in a high-throughput manner [1]. High-throughput datasets have been generated for model organisms such as yeast [2-6], worm [7] and fly [8,9] as well as Escherichia coli [10]. In addition, the first broad-focus experimental datasets for the human interactome have recently been published [11,12]. Interactions determined by high-throughput methods are generally considered to be less reliable than those obtained by low-throughput studies [13,14] and as a consequence efforts are also underway to extract evidence for interactions from the literature [15-17]. Analysis of the high-throughput datasets has shown that they overlap very little with each other, suggesting that their coverage is low. Indeed, it has been estimated recently that the current yeast and human protein interaction maps are only 50% and 10% complete, respectively [18].

The low coverage and variable quality of the experimental interaction datasets have prompted many groups to investigate computational methods to predict interactions or to determine the most likely interactions seen in the high-throughput datasets. The different approaches to predict interactions can be grouped into five main categories:

1) Predictors based on sequence and structure exploit the observation that some pairs of sequence motifs, domains and structural families tend to interact preferentially. Some methods predict interaction from sequence-motifs found to be over-represented in interacting protein pairs [19], or by considering the physico-chemical properties and the location of groups of amino acids in the sequence [20,21]. Others investigate the co-occurrence in interacting proteins of specific protein domains or their structural family classification [22,23]. When three-dimensional structures are available for both proteins thought to interact, high quality predictions and additional information such as the residues involved in the interaction and their binding affinity may be estimated (reviewed in [24]). Similarly, when two proteins show clear sequence similarity to proteins that exist in a complex for which the three-dimensional structure is known, detailed predictions of the atomic-level interactions may be made. For example, the major complexes in yeast have been predicted by this strategy [25].

2) Predictors based on comparative genomics have been exploited primarily in prokaryotes. They consider the physical location of genes, as well as their pattern of occurrence and evolutionary rate, to predict interactions or functional relationships between protein pairs. Some predictors make use of the observation that neighboring genes whose relative location is conserved across several prokaryotic organisms are likely to interact [26]. Other predictors exploit the observation that gene pairs that co-occur in related species or that co-evolve also tend to be more likely to interact [27-30]. In addition, domains that exist as separate proteins in some genomes but are also seen fused in a single protein in other genomes have been used to suggest the isolated domains may interact [31,32].

3) Predictors based on orthology work on the assumption that the orthologs of a protein pair that are known to interact in one organism will also interact. Such relationships are often referred to as interologs [33]. For example, at BLAST e-values below 10^-10^, it has been shown that 16–30% of yeast interactions can be transferred to the worm [34] while further studies have estimated that a joint e-value below 10^-70 ^is required to transfer interactions reliably between organisms [35]. Interologs have been used to predict protein-protein interactions in human [36].

4) Predictors based on functional features exploit non-sequence information to infer interactions. Some predictors exploit the observation that there is a significant correlation in the expression levels of transcripts encoding proteins that interact [37]. Since proteins must be co-localized in order to interact, protein subcellular localization has often been used to assess the quality of interaction datasets [38,39]. Similarly, interacting proteins are also often involved in similar cellular processes, so Gene Ontology "process" and "function" annotations have been exploited to predict interactions and validate high-throughput datasets [16,36,38].

5) Predictors have exploited similarities in the network topology of known interaction datasets to predict novel interactions. In one study, the local topology of small-world networks has been used to assess the quality of interaction datasets and predict novel interactions [40] while Gerstein and colleagues have investigated the prediction of interactions by the identification of missing edges in almost fully connected complexes [41].

In addition to these diverse approaches, some groups have combined concepts from several of the above categories in integrative frameworks. The first such predictor integrated co-expression data, co-essentiality as well as biological function in a naïve Bayes network to provide proteome-wide *de novo *prediction of yeast protein interactions [37]. Subsequently, the combination of many more diverse features was investigated using different frameworks to predict yeast protein-protein interactions, increasing the prediction accuracy and allowing an assessment of the limits of genomic integration [42-44]. The integration of diverse genomic features has also been useful in the investigation of the related but broader problem of predicting protein-protein associations as well as complex and pathway membership (see for example [45]).

Although, many computational methods have investigated the prediction of protein-protein interactions, few have so far been applied to the human proteome. The first large-scale prediction of the human interactome map involved transferring interactions from model organisms [36]. This resulted in over 70000 predicted physical interactions involving approximately 6200 human proteins. A second method integrated expression data, orthology, protein domain data and functional annotations into a probabilistic framework and resulted in the prediction of nearly 40000 human protein interactions [46]. It has recently been estimated that the false-positive rates of these computational datasets as well as of available high-throughput human interaction datasets are, on average, as high as 90% and their coverage is only approximately 10%, indicating that more such efforts are needed to increase the coverage and confidence we have in current maps of the human interactome [18].

In this paper, the prediction of physical interactions between human proteins has been investigated by integrating in a Bayesian framework several different pieces of evidence including orthology, functional features and local network topology. In order to increase the accuracy and coverage of the predictions, different types of negative data (non-interacting protein pairs) were explored to train the predictor. The most accurate of the predictors was then used to assess the likelihood of pair-wise interaction for over 20000 human proteins from the IPI (International Protein Index) database. These predictions provide a likelihood of interaction for over 260 million human protein pairs and lead to the prediction of over 37000 human interactions. They should thus augment current knowledge of the human interactome as well as the understanding of the relationship between distinct cellular processes.

## Results and discussion

### Architecture of the predictor and training of the modules

**Figure 1:**
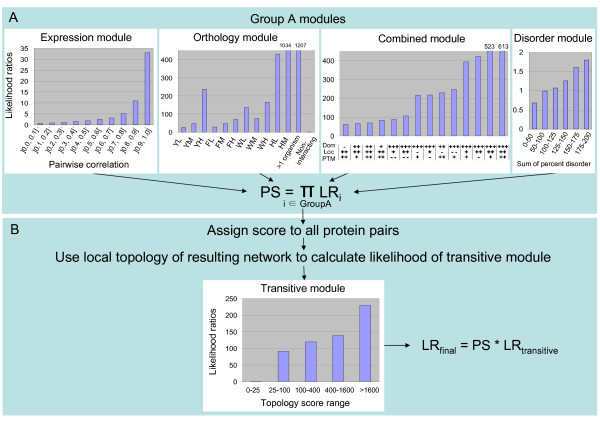
Architecture of the predictor and likelihoods of the modules. The predictor consists of two different parts (A and B) which are trained consecutively. The Group A modules (shown in panel A) are trained in parallel. The likelihood ratios (LR) for most of their states are shown in panel A (their complete likelihood ratios are available in Additional File 4). The product of the likelihood ratios of all Group A modules considered in a given prediction is referred to as the preliminary score (PS) and can be calculated for all human protein pairs. If the Transitive module is not considered, the final likelihood ratios assigned to all protein pairs is the preliminary score (PS). If the Transitive module is considered, the local topology of the network determined by the assignment of preliminary scores to all protein pairs considered in the training set is used to calculate the likelihood ratios for the transitive module (shown in panel B) for every protein pair in the training set. The final likelihood ratio is then the product of the preliminary score calculated in panel A and the likelihood ratio output by the transitive module in panel B. For the Orthology module: YL, YM, YH: yeast low, medium and high scoring bins; FL, FM, FH: fly low, medium and high scoring bins; WL, WM, WH: worm low, medium and high scoring bins; HM and HL: medium and low scoring bins for human protein pairs that have human paralogs; > 1 organism: bin for human protein pairs that have interologs in more than one organism. For the Combined module, –— refers to the lowest scoring bin (for the domain (Dom), post-translational modification (PTM) and subcellular localization (Loc) features), – refers to the second lowest scoring bin and +, ++, +++ refer respectively to the third highest, second highest and highest scoring bins.

The prediction of human protein-protein interactions was investigated in a Bayesian framework by considering combinations of individual protein features known to be indicative of interaction. The seven individual features considered are summarized in Table 1 and detailed in the Methods section. As indicated in Table 1, the different features were grouped into five distinct modules: Expression (E), Orthology (O), Combined (C), Disorder (D) and Transitive (T). Figure 1 illustrates the training scheme and architecture of the method. The Expression, Orthology, Combined and Disorder modules can calculate likelihood ratios (LR) of interaction independently and are referred to as the Group A modules (Figure 1A). The product of their likelihood ratios is referred to as the Preliminary Score. The Transitive module considers the local topology of the network predicted by the group A modules and thus requires the completion of their analysis to calculate its own likelihood ratios of interaction (Figure 1B). As such, all combinations of the Group A modules can be used to predict interaction in the presence or absence of the Transitive module. In the absence of the Transitive module, the Preliminary Score is used as the final likelihood ratio output by the predictor.

**Table 1:** Features considered in the prediction of interactions for each module

**Module abbreviation**	**Features considered**	**Data source**	**Description**	**Scoring function**	**Bins**
**E**	Expression	GDS596 from the Gene Expression Omnibus [70]	Gene expression profiles from 79 physiologically normal tissues obtained from various sources [71]	Pearson correlation of co-expression over all conditions	20 of equal size covering the correlation value range (-1 to +1)

**O**	Orthology	InParanoid [72], BIND [66], DIP [65] and GRID [69] databases	Interactions of homologous protein pairs from yeast, fly, worm and human	Organism-based using InParanoid score	13

**C (Combined)**	Localization	PSLT predictions [54]	PSLT is a human subcellular localization predictor that considers nine different compartments (ER, Golgi, cytosol, nucleus, peroxisome, plasma membrane, lysosome, mitochondria and extracellular)	Qualitative score: proximity of compartments	4 (same, neighboring, different compartments, or not localized)
	
	Domain co-occurrence	InterPro [73] and Pfam [74]	Protein domains and motifs	Chi-square	5 covering range of Chi-square scores
	
	PTM co-occurrence	HPRD [15] and UniProt [76]	Post-translational modifications	P(PTM[i],PTM[j]|I)P(PTM[i]|I)∗P(PTM[j]|I) MathType@MTEF@5@5@+=feaafiart1ev1aaatCvAUfKttLearuWrP9MDH5MBPbIqV92AaeXatLxBI9gBaebbnrfifHhDYfgasaacH8akY=wiFfYdH8Gipec8Eeeu0xXdbba9frFj0=OqFfea0dXdd9vqai=hGuQ8kuc9pgc9s8qqaq=dirpe0xb9q8qiLsFr0=vr0=vr0dc8meaabaqaciaacaGaaeqabaqabeGadaaakeaadaWcaaqaaiabbcfaqjabcIcaOiabbcfaqjabbsfaujabb2eanjabcUfaBjabbMgaPjabc2faDjabcYcaSiabbcfaqjabbsfaujabb2eanjabcUfaBjabbQgaQjabc2faDjabcYha8jabbMeajjabcMcaPaqaaiabbcfaqjabcIcaOiabbcfaqjabbsfaujabb2eanjabcUfaBjabbMgaPjabc2faDjabcYha8jabbMeajjabcMcaPiabgEHiQiabbcfaqjabcIcaOiabbcfaqjabbsfaujabb2eanjabcUfaBjabbQgaQjabc2faDjabcYha8jabbMeajjabcMcaPaaaaaa@5C25@	4 covering range of PTM scores

**D**	Disorder	VLS2 predictions [78]	Prediction of protein intrinsic disorder	Sum of the percent disorder for each protein in a pair	6 covering range of scoring function (0 to 200%)

**T**	Transitive	-	Module that considers local topology of underlying network predicted using combinations of above features	T=∑e∈Ecse1+|Ei\Ec|+|Ej\Ec| MathType@MTEF@5@5@+=feaafiart1ev1aaatCvAUfKttLearuWrP9MDH5MBPbIqV92AaeXatLxBI9gBaebbnrfifHhDYfgasaacH8akY=wiFfYdH8Gipec8Eeeu0xXdbba9frFj0=OqFfea0dXdd9vqai=hGuQ8kuc9pgc9s8qqaq=dirpe0xb9q8qiLsFr0=vr0=vr0dc8meaabaqaciaacaGaaeqabaqabeGadaaakeaacqqGubavcqGH9aqpdaWcaaqaamaaqafabaGaee4Cam3aaSbaaSqaaiabbwgaLbqabaaabaGaeeyzauMaeyicI4Saeeyrau0aaSbaaWqaaiabbogaJbqabaaaleqaniabggHiLdaakeaacqaIXaqmcqGHRaWkcqGG8baFcqqGfbqrdaWgaaWcbaGaeeyAaKgabeaakiabcYfaCjabbweafnaaBaaaleaacqqGJbWyaeqaaOGaeiiFaWNaey4kaSIaeiiFaWNaeeyrau0aaSbaaSqaaiabbQgaQbqabaGccqGGCbaxcqqGfbqrdaWgaaWcbaGaee4yamgabeaakiabcYha8baaaaa@4F06@	5 covering range of scoring function

The likelihood ratios of interaction are evaluated for each module by considering the relative proportions of positive and negative training examples that have a specific state (i.e. that fall in a particular bin of a module). The datasets used to train the predictor consisted of 26896 known human protein interactions extracted from the Human Protein Reference Database (HPRD) [15] and approximately 100 times more randomly chosen protein pairs used as negative examples. The composition of the datasets and likelihood ratio calculations are explained in greater detail in the Methods section. Once the final likelihood ratio of interaction (LR_final_) is calculated for a given protein pair as shown in Figure 1B, it is possible to estimate the posterior odds ratio of interaction by multiplying the final likelihood ratio by the prior odds ratio of interaction. Protein pairs that have a posterior odds of interaction above 1 are more likely to interact than not to interact, thus providing an obvious threshold to predict interacting proteins. Estimates for the prior odds ratio of interaction vary. Previous interaction studies on yeast and human use prior odds ratios that range from 1/600 to > 1/400 [37,43,46,47]. The evaluation of this ratio is difficult because not all true interactions are known. As detailed in Methods, the prior odds ratio for human protein interaction was explored by considering different versions and subsets of human interaction datasets. This suggested that there is insufficient data currently available to determine a reliable ratio for human. Accordingly, we selected a prior odds ratio of interaction of 1/400 which is similar to current estimates for yeast and is probably still quite conservative. Thus, the likelihood ratio threshold to predict interactions is 400.

### Likelihood ratios of the modules

Figure 1 summarizes the likelihood ratios computed for the five modules. The different modules differ in the range of likelihood ratio values achieved by their different states. The Orthology and Combined modules both have states that achieve likelihood ratios above 400 (as high as 1207 for the Orthology module and 613 for the Combined module), indicating that both these modules can, on their own, predict some interacting protein pairs with a posterior odds ratio above 1.

The Expression module follows trends seen in previous studies with increasing likelihood ratios of interaction reflecting increasing expression correlation [37,46]. However, since the highest likelihood ratio for the expression datasets that we consider is 33, they are not sufficient on their own to predict interacting protein pairs with a posterior odds ratio above 1. Similarly, but in a much more pronounced way, the Disorder module is only slightly predictive of interaction, with a maximum likelihood ratio of 1.8.

Most states of the Orthology module achieve higher likelihood ratios than the highest obtained by the Expression and Disorder modules. This is not surprising as the transfer of interacting orthologs (known as interologs [33]) from one organism to another is a popular method to predict interactions (see for example [34,48]), particularly in the case of organisms like human for which only a small proportion of interactions are known. The direct transfer of interactions to human from either yeast, fly or worm does not alone result in a posterior odds ratio above 1 (as the likelihood ratios of interaction for all yeast, fly and worm bins in the Orthology module are below 400). This is not surprising as previous studies have indicated that quite stringent joint E-values must be used to transfer interactions safely between organisms [34,35]. In contrast, the consideration of human interactions paralogous to the human protein pairs under investigation results in likelihood ratios of 431 and 1034 (depending on how close the paralogs are as described in Methods) which is much higher than those obtained for any single model organism. This agrees with a recent report that suggested protein-protein interactions are more conserved within species than across species [49].

**Figure 3:**
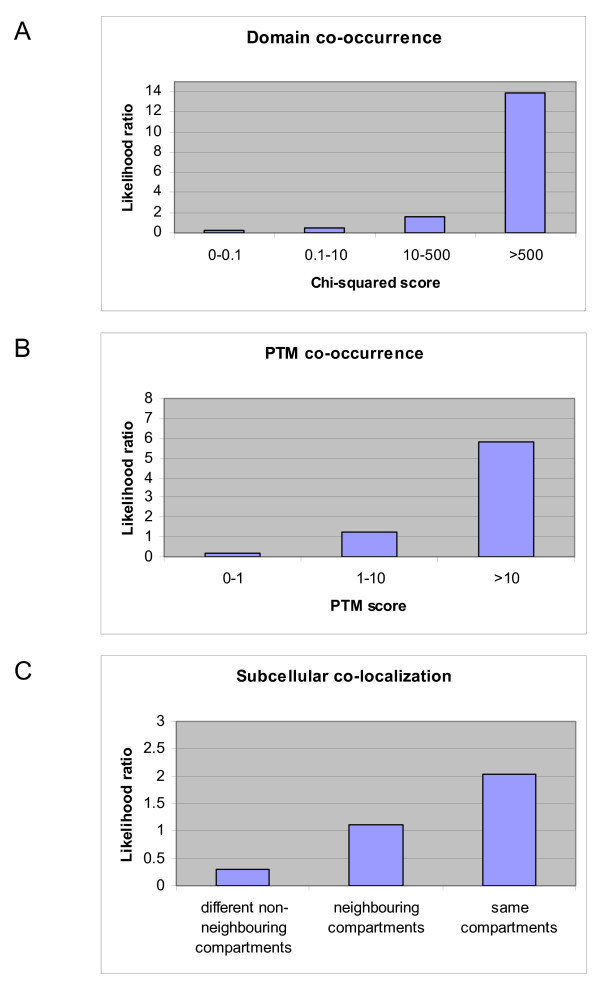
Likelihood ratios of the features that form the Combined module, considered separately. The Combined module considers simultaneously three distinct features: the co-occurrence of both domains and PTMs as well as the subcellular co-localization of proteins. Here the likelihood ratios of these features considered separately are shown. In panel A, all domain pairs considered were given scores and likelihood ratios were estimated for different values of these scores. Similarly, shown in panel B are the likelihood ratios for different values of PTM co-occurrence scores. Panel C shows the likelihood ratios for protein pairs localized to different sets of cellular compartments.

The Combined module uses domain co-occurrence, post-translational modification (PTM) co-occurrence and subcellular localization information to predict interaction. These features were originally investigated separately, as shown in Figure 3, but their combination into one module that considers all dependencies between them achieves higher accuracy (data not shown) and higher likelihood ratios (as can be seen by comparing to Figure 1) while still being computationally feasible. Additionally, this combination circumvents possible problems of dependence between these features.

Previous methods have investigated the use of co-occurring domains to predict interaction (see for example [23,46]). Many pairs of domains co-occur in proteins known to interact. When investigated as a separate feature, the chi-square score of co-occurrence of domain pairs correlates well with the likelihood of interaction of protein pairs that contain these domains, with the highest chi-square score bin obtaining a likelihood ratio of 14, as shown in Figure 3A. Similarly, the co-occurrence of PTMs is also predictive of interaction, with its highest scoring bin obtaining a likelihood ratio of 6 as shown in Figure 3B. Lists of high scoring domain pairs and PTM pairs are shown in Additional Files 1 and 2.

Subcellular localization has been extensively used both to assess the quality of interaction datasets [11,50,51] and to generate examples of non-interacting protein pairs to use as negative datasets when training and testing predictors [37,46]. In the present study, the use of localization was investigated as a feature predictive of interaction. Four possible localization states were considered for protein pairs: same compartment, neighboring compartments, different non-neighboring compartments and absence of localization annotation (more details are given in the Methods section). As shown in Figure 3C, the likelihood ratio of same compartment protein pairs was found to be twice as high as that of randomly chosen or non-annotated protein pairs whereas different non-neighboring protein pairs are more than three times less likely to interact than random protein pairs Individual localization features achieve low interaction likelihood ratios. However, when integrated into the Combined module, domain, PTM and localization information together achieve likelihood ratios that are high enough to predict interaction on their own (i.e. above 400). As expected, the highest likelihood ratio bins for the Combined module are those representing the highest combinations of the three features separately.

The transitive module enhances the preliminary likelihood score (PS) (calculated using the group A modules) by considering the local topology of the resulting network which is assessed using the neighborhood topology score as detailed in the Methods section. The likelihood ratios for different values of the neighborhood topology score are shown in Figure 1B. The Transitive module is highly predictive of interaction and achieves likelihood ratios as high as 229. This module cannot be used alone as it requires as input the output of at least one group A module. However, it can predict interacting protein pairs with a posterior odds ratio above 1.0 when used in combination with any single module in group A (as the product of the highest likelihood ratios of the transitive module and any group A module is greater than 400 as can be seen from Figure 1).

### Independence of the modules

The final likelihood ratio output by the predictor is only representative of the true likelihood of interaction of a protein pair if the modules considered are independent. If the modules were not independent, some likelihood ratios would likely be overestimated, particularly for protein pairs that achieve simultaneously high likelihoods for non-independent features. Conversely, some likelihood ratios would be underestimated for protein pairs achieving simultaneously low likelihoods for non-independent features. Previous studies have demonstrated that some of the features considered here are indeed independent [43]. Independence of all modules used in our predictor was verified by calculating Pearson correlation coefficients for all pairs of modules. As shown in Table 2, all modules considered are independent, since the highest Pearson correlation coefficients computed are well below any value considered significant.

**Table 2:** Pairwise Pearson correlation for all modules

	Expression	Orthology	Combined	Disorder	Transitive
Expression	-	0.00460	0.01299	0.00995	0.00562
Orthology	-	-	0.01000	0.00977	0.01555
Combined	-	-	-	0.02086	0.02380
Disorder	-	-	-	-	-0.01702
Transitive	-	-	-	-	-

### Accuracy of the predictors

All combinations of modules were examined to determine which of the resulting predictors achieved the highest prediction accuracy. In order to analyze the predictions, five-fold cross validation experiments were performed and the area under partial ROC (receiver operator characteristic) curves (partial AUCs) measured. ROC50 and ROC100 curves were selected as they consider a large enough number of positives to include all protein pairs predicted to have a posterior odds ratio above 1.0 by all the predictors investigated. Protein pairs predicted to have a posterior odds ratio below 1.0 have an estimated true positive rate below 50% and thus are more likely not to interact than to interact. These protein pairs are therefore not of interest in this context. The area under all ROCn curves considered is relatively low because of the high proportion of negatives with respect to positives in the training and test sets (100:1).

Table 3 summarizes the characteristics of 19 different predictors and shows accuracy measures. Individual modules do not achieve high scores for the areas under the ROC50 and ROC100. In fact, all ROC50 AUC values achieved by individual modules are below 0.025 and the Expression and Disorder modules do not predict any protein pairs (positive or negative) above a posterior odds ratio of 1, which is expected as the highest likelihood ratios they achieve are lower than 400 (see Figure 1A). As more Group A modules are considered within the same predictor, the ROCn AUC scores increase significantly, as would be expected since these features are independent (as shown in Table 2) and thus contribute different information to the prediction. For example, the predictor that considers both the Expression and Combined modules achieves a ROC50 AUC of 0.033 compared to 0.003 and 0.022 respectively for the individual modules. However, the Disorder module does not contribute significantly to the prediction as predictors that consider it do not, in general, do better than their counterparts that do not use it. For example, both the Expression-Orthology predictor and the Expression-Orthology-Disorder predictor achieve a ROC50 AUC of 0.024. The Disorder module offers the advantage of increasing the coverage of the prediction as a disorder score is calculated for all protein pairs. However, this appears to add more noise to the prediction without increasing the accuracy.

**Table 3:** Prediction accuracy of different combinations of modules

**Modules included in prediction**																			
Expression	•				•	•	•				•	•	•		•		•		•
Ortho		•			•			•	•		•		•	•	•	•	•	•	•
Combined			•			•		•		•	•	•		•	•	•	•	•	•
Disorder				•			•		•	•		•	•	•	•			•	•
Transitive																•	•	•	•

**Coverage of the Informative Protein Set (%)**																			

	22	23	32	99	40	43	99	49	99	99	57	99	99	99	99	90	95	99	99

**Measures of accuracy**																			

ROC50 AUC	0.003	0.018	0.022	0	0.024	0.033	0.003	0.042	0.021	0.030	0.044	0.031	0.024	0.038	0.042	0.071	**0.075**	0.069	0.072
ROC100 AUC	0.003	0.026	0.032	0	0.030	0.045	0.005	0.054	0.027	0.041	0.058	0.044	0.029	0.049	0.058	0.090	**0.094**	0.088	0.093

**Estimation of number of interactions predicted**																			

posterior odds ratio > 4	0	420	0	0	1050	630	0	2520	0	0	3780	630	0	2888	2520	14200	16590	13400	**16800**
posterior odds ratio > 2	0	630	1050	0	2520	2100	0	3780	210	2100	7980	2100	1050	4200	5460	21340	**24570**	21200	24200
posterior odds ratio > 1	0	840	4830	0	5670	7140	0	11760	1890	4200	15330	5460	3990	13125	13860	28500	**34780**	28600	33180

As the scores of the predictors increase, so do the number of interactions predicted above different posterior odds ratio thresholds (see lower portion of Table 3). For example, the Expression-Orthology predictor achieves a ROC50 AUC of 0.024 and predicts 5670 interactions at a posterior odds ratio greater than 1 whereas the Expression-Orthology-Combined predictor achieves a ROC50 AUC of 0.044 and predicts over 15000 interactions at a posterior odds ratio above 1. The best combination of Group A modules is the predictor consisting of the Expression, Orthology and Combined modules.

The Transitive module, which can only be used in combination with other modules, increases substantially the scores and number of interactions predicted. The right-hand portion of Table 3 shows the accuracy measures for the highest scoring subset of predictors that consider the Transitive module. The Transitive module enhances the prediction by identifying among protein pairs with a relatively high preliminary score those that are most likely to interact, by considering the local topology of the network around them. For example, the ROC50 AUC rises from 0.044 to 0.075 when the Transitive module is added to the Expression-Orthology-Combined predictor, and the number of predictions above a posterior odds ratio of 1 doubles from 15330 to 34780. Once again, the Disorder module does not contribute positively to the prediction. Its inclusion does not increase any of the measures of accuracy considered. The predictor that considers the Expression, Orthology, Combined and Transitive modules is the one that achieves the highest accuracy overall. It is this predictor that is further analyzed in the next sections.

### Comparison to predictions generated using alternative training sets

In this work training sets were used that comprised 100 times more negatives than positives, with the negatives randomly selected and filtered to remove any known or suspected positives (see Methods). Other groups have used negative:positive ratios ranging from 1 to more than 600 (see for example [37,47,52]). In addition, several groups use localization-derived negatives (i.e. protein pairs that are not annotated as being localized to the same cellular compartment) rather than randomly chosen negatives (see for example [37,43,46]). These issues have been investigated previously [53].

Since the choice of negative training data may influence the method, the choice of different training sets in the context of the probabilistic predictor presented here was investigated to determine which type of training set offers the highest accuracy.

Table 4 compares the accuracy of predictors trained with negative:positive ratios of 1:100 and 1:1 and tested by five-fold cross validation. Ratios greater than 100 were not considered because they are computationally infeasible given the size of our datasets and the architecture of the predictor. To perform such a comparison, the EOCT predictor (Expression, Orthology, Combined and Transitive modules) was trained on datasets consisting of either equal numbers of positives and negatives or 100 times more negatives than positives and then tested on both types of datasets. As shown in Table 4, the predictors trained on datasets containing 100 times more negatives than positives perform significantly better than those trained on datasets containing equal numbers of positives and negatives. For example, the 1:1 pos:neg trained predictor achieves a ROC50 AUC of 0.0645 whereas its 1:100 pos:neg trained counterpart achieves a 0.0747 ROC50 AUC. This could be due to the fact that the number of non-interacting protein pairs outweighs greatly the number of interacting protein pairs in cells. When equal numbers of positives and negatives are used in training, the diversity that exists in the non-interacting protein pair space may not be captured, thus resulting in misleading likelihood ratios for the predictive modules. It should be noted that predictors tested on datasets consisting of equal numbers of positives and negatives achieve much higher accuracy measures than those tested on datasets containing 100 times more negatives than positives. This is because the number of positives scoring higher than the highest scoring n negatives, for a given value of n and a given predictor, will be greater if there are equal numbers of positives and negatives in the test set than if there are more negatives than positives.

**Table 4:** Influence of the negative:positive training set ratio on the prediction accuracy

		Neg:pos testing ratio			
		
		1:1	1:1	100:1	100:1
		
		ROC50 AUC (std)^a^	ROC100 AUC (std)^a^	ROC50 AUC (std)^a^	ROC100 AUC (std)^a^
Neg:pos training ratio	1:1	0.300 (0.008)	0.385 (0.006)	0.0645 (0.0019)	0.0814 (0.0009)
	100:1	0.325 (0.004)	0.403 (0.003)	0.0747 (0.0022)	0.0944 (0.0028)

^a ^The ROCn AUCs are an average of five separate experiments (each of which is itself a five-fold cross validation experiment). Their standard deviation is shown in parenthesis.

The effect of localization-derived negatives rather than randomly chosen negatives was also investigated to see if it would increase the prediction accuracy. A criticism of randomly chosen negatives is that they will contain some true interactors. However, the set of interacting pairs in the full protein pair space is small and thus the contamination rate of randomly chosen negative datasets will in fact be very low. Contamination is probably below 1%, which is likely lower than the contamination rate of the positive dataset as discussed in [47]. Localization-derived negatives, on the other hand, should be free of contamination, if the localization annotations are complete and accurate, both conditions that are difficult to obtain as discussed in [54]. However, one can argue that localization-derived negatives might not be able to capture the full diversity of the non-interacting protein space since many proteins in the same cellular compartment do not interact. In addition, proteins specific to a cellular compartment may have different characteristics to proteins in other compartments. Such predictors may not generalize well when predicting on cell-wide protein pairs which consist not only of non-colocalized non-interacting pairs but also numerous protein pairs that do not interact but are present in the same cellular compartment. These issues have been discussed previously [52]. In order to see if different types of negatives could influence the accuracy of the predictors developed here we generated negative training/test sets as in [46] by identifying all pairs of human proteins for which one protein is annotated as being nuclear and the other is annotated as being localized to the plasma membrane in the HPRD database [15]. The Combined module for these predictors only considers domains and PTMs but not subcellular localization as this would result in using this feature both in the selection of the training set and as a feature predictive of interaction. The localization-derived negative trained predictor tested on sets containing localization-derived negatives achieves a lower accuracy than that of the random negative trained predictor tested on a test set containing randomly-generated negatives (0.0686 +/- 0.0010 vs 0.0747 +/- 0.0022). This is most likely due to the fact that the localization-derived negative trained predictor cannot take full advantage of the Transitive module, since the network resulting from the predictions of the Group A modules likely does not sample the whole protein pair space well.

Our predictor trained with randomly generated negatives and a negative:positive ratio of 100 performs the best out of all the combinations of training sets investigated. It is this predictor that is further analyzed in subsequent sections.

### Contribution of the modules

The relative contribution of the modules to the prediction of interaction was investigated in order to gain a better understanding of the predictive power and areas of highest usefulness of the different modules. To do this, all protein pairs were considered that achieve an estimated posterior odds ratio > 1 when the EOCT predictor was trained on the full datasets without cross-validation. This set consists of 37606 distinct predicted interactions and is referred to as the LR400 dataset (all these interactions are listed and ranked in Additional File 3). These protein pairs represent the most probable interactors with respect to the features considered, among all protein pairs examined by the predictor.

**Figure 4:**
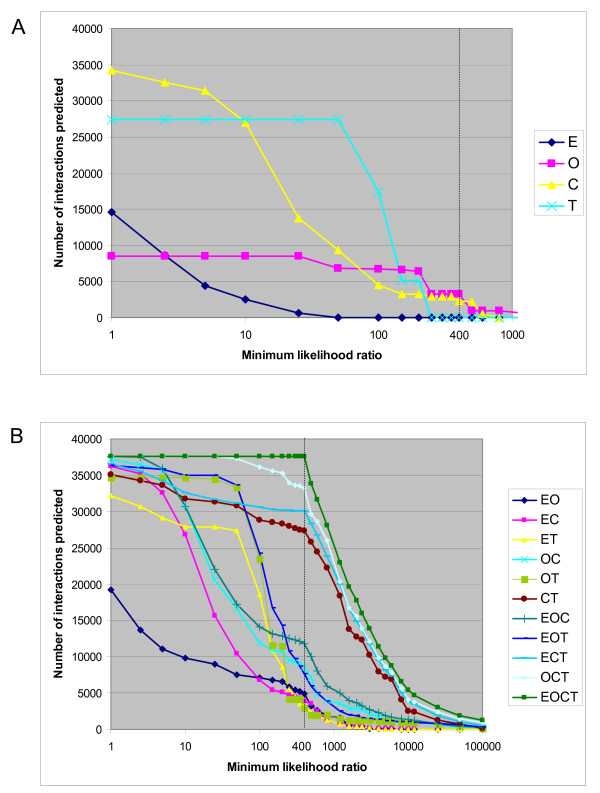
Contribution of the modules. To examine the contribution of the different modules, we plotted the number of interactions predicted among all LR400 interactions (all interactions predicted using the full predictor that obtain a likelihood ratio of interaction greater than 400) as a function of the minimum likelihood ratio of individual modules (in panel A) or of combinations of modules (in panel B). In the case of combinations of modules (panel B), the minimum likelihood ratio is the product of the likelihood ratios of the modules considered. Thus for example, the product of the expression and orthology ratios is greater than 1 for almost 20000 LR400 interactions and greater than 10 for approximately 10000 LR400 interactions (dark blue diamonds in panel B). E: Expression module, O: Orthology module, C: Combined module, T: Transitive module.

To investigate the individual contribution of each module, we looked at the number of interactions predicted out of all LR400 pairs as a function of the minimum likelihood ratio of each module. As shown in Figure 4A, all modules contribute positively (i.e. contribute a likelihood ratio greater than 1.0) to the prediction of a certain proportion of the interactions in the LR400 dataset. The Transitive module and to an even greater extent, the Combined module contribute positively to the prediction of a very high proportion of the LR400 protein pairs (73% and 91% of the LR400 interactions have likelihood ratios greater than 1 for the Transitive and Combined modules respectively). The Transitive module provides a likelihood ratio of 91 for the prediction of over 70% of the LR400 interactions. The Combined module provides positive evidence for the highest number of interactions of the LR400 dataset. However, the value of the likelihood ratio it contributes is below 20 for over 50% of protein pairs in the LR400 dataset (which means that for these protein pairs, the Combined module must be used in combination with other modules to achieve a total likelihood ratio above 400). The Combined module does, however, achieve likelihood ratios high enough to predict over two thousand interactions of the LR400 dataset on its own, less than 15% of which are present in the training set. The Orthology module contributes to the prediction of only 8474 protein pairs in the LR400 dataset (23%). However, a large majority (> 75%) of these 8474 predicted interactions achieve likelihood ratios above 200 from this module. In fact, almost 40% of these LR400 interactions achieve a likelihood ratio above 400 from the Orthology module. This indicates that most interactions predicted by the Orthology module (alone or in combination with other modules) are based on the highest scoring Orthology bins (see Figure 1A) which are the most conserved yeast interactions (whose bin achieves a likelihood ratio of 237), as well as human paralogous interactions and interactions found in more than one model organism (both of which achieve a likelihood well above 400). Few interactions in the LR400 dataset are predicted on the basis of having interacting orthologs in worm or fly alone. The Expression module provides positive evidence for a little less than half the predictions in the LR400 dataset. However, as previously noted, the highest likelihood provided by this module is 33 and thus the Expression module cannot predict interaction on its own.

**Figure 5:**
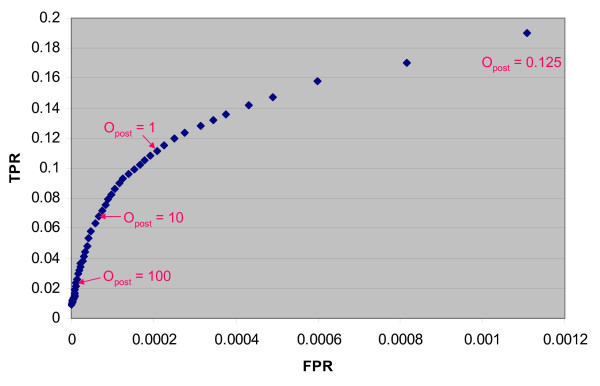
True positive rate versus false positive rate for different estimated posterior odds ratios. The true positive rate (TPR) versus false positive rate (FPR) is plotted for different values of the posterior odds ratio estimated for the dataset by five-fold cross-validation. As the posterior odds ratio increases, the false positive rate decreases and the ratio of the true positive rate divided by the false positive ratio increases. Thus, higher quality datasets can be generated by requiring higher posterior odds ratios. The TPR is calculated as the number of true positives predicted divided by the total number of positives in the test set. The FPR is calculated as the number of false positives predicted divided by the total number of negatives in the test set.

Figure 4B summarizes the contributions of different combinations of modules. The Combined and Transitive modules contribute the most to the prediction of interactions. They alone can predict approximately 27000 of the 37606 interactions of the LR400 dataset. When they are both present, regardless of which other modules are also present, they predict over 70% of the LR400 interactions. When either of these two modules is absent, fewer than 12500 interactions are predicted. In contrast, the two remaining modules (Expression and Orthology) can predict approximately 5000 interactions together. This is interesting as many of the publicly available predicted interaction datasets mentioned in the Background section use mainly orthology transfer from model organisms to identify interactions. As the majority of the LR400 interactions are derived from the Combined and Transitive modules, it is possible that the method is identifying a large subset of interactions that are not common to previous human protein interaction datasets. This is discussed further in the next section. The curve representing the full predictor (consisting of the Expression, Orthology, Combined and Transitive modules) is also represented in Figure 4B (the dark green squares). By definition, it predicts all proteins in the LR400 dataset at likelihood ratios equal to or above 400 (this is how the LR400 dataset was generated). The right side of the curve illustrates the number of interactions that are predicted above likelihood ratios of 400 and more. As shown in Figure 4B, the full predictor predicts approximately 20000 interactions at a total likelihood ratio of 1600 (which is equivalent to an estimated posterior odds ratio of 4). At a likelihood ratio of 4000, approximately 11000 interactions are predicted and at a likelihood ratio of 8000, approximately 6500 interactions are predicted. We verified that the increasing estimated posterior odds ratios translated into better predictive value. Figure 5 shows the true positive rate versus false positive rate for different posterior odds ratios as measured by five-fold cross validation. As the posterior odds ratio increases, the false positive rate decreases and the relative proportion of true positives increases when compared to the proportion of false positives. Accordingly, subsets of very high quality predictions may be generated by choosing a suitably high posterior odds ratio threshold.

### Comparison to other interaction datasets

**Figure 6:**
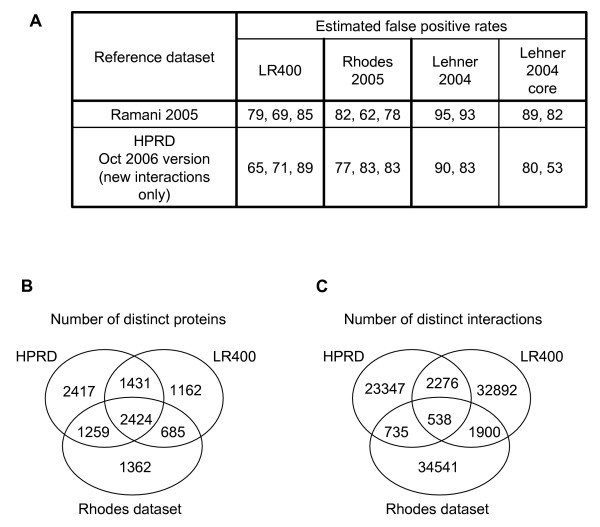
Comparison to other interaction datasets. The false positive rates shown in panel A were estimated for the LR400 dataset as well as the Rhodes [46] and Lehner [36] predictions using the method described in [18, 55] by comparing them two-by-two to a reference dataset. The number and overlap of distinct proteins (shown in B) and distinct interactions (shown in C) are shown for the LR400 dataset, the Rhodes prediction dataset and the June 2006 version of the HPRD.

The false positive rate (FPR) of our predictor was estimated by the method of D'Haeseleer and Church [18,55] and used to compare it to other prediction datasets. The Ramani interaction dataset that was automatically extracted from the literature [16] as well as all new interactions present in the October 2006 version of the manually curated HPRD database [15] (but none of the interactions also present in earlier versions of the HPRD which were used to train our predictor) were taken as reference datasets. The D'Haeseleer and Church method compares two experimental datasets to a reference set and assumes that all intersections between the three datasets contain true positives. It is thus possible to estimate the number of true positives predicted by an experimental dataset by comparing the number of interactions present in the different intersections of the two experimental methods and the reference dataset (for details, see [18,55]). Here, we compare three human interaction prediction datasets: the Rhodes probabilistic dataset [46], the Lehner orthology-derived dataset [36] and the most accurate of our predictors (the LR400 subset of the predictor considering the Expression, Orthology, Combined and Transitive modules). We estimated false positive rates for each of the datasets by comparing them two by two to one of the reference datasets, thus generating 4 to 6 different estimates of false positive rates for each computational dataset, as shown in Figure 6A (the two Lehner datasets were not compared to each other, which is why they have fewer FPR estimates). The rates estimated for the Rhodes and Lehner datasets are similar to previous estimates [18]. The estimated false positive rates for the LR400, Rhodes and core Lehner are quite similar (an average of 76% FPR for both the LR400 and core Lehner datasets and 78% for the Rhodes dataset) and well below the overall average false-positive rate of 90% estimated for most available human high-throughput experimental and prediction interaction datasets [18]. It should be noted that the Rhodes, Lehner and Ramani datasets annotate interactions as a relationship between human genes and not their protein products directly. However, not all proteins encoded by a single gene will necessarily interact with all protein products encoded by a second gene, even if one such protein pair does. This is why we describe interactions as a relationship between two proteins, allowing for a more precise description of the interaction. To compare our predictions to these datasets, we consider that two genes interact if at least one of their respective protein products interact.

In Figure 6B and 6C, we compare the number of distinct proteins and distinct interactions of the LR400 dataset to those of the Rhodes prediction dataset and the June 2006 version of the HPRD which was used to train our predictor. The Rhodes dataset was trained using an earlier version of the HPRD. As can be seen in Figure 6, the intersections between the three datasets considered are low, especially when comparing the interactions. Both the Rhodes dataset and our LR400 dataset predict interactions involving many proteins that are not even present in their positive training set (the HPRD). Many of the predictions in these two datasets concern protein pairs and proteins that are not present in other datasets, suggesting that they cover different regions of the human interaction space. As suggested in [18], by making more such datasets available, it will be possible to increase our coverage of the interaction space and determine the most likely human interactions.

Another human interaction dataset has recently become available: the IntNetDB [56]. It was generated by integrating seven different features (four of which involve transferring interactions or characteristics of protein pairs from model organisms to human) in a probabilistic framework. Interactions were predicted above a TP/FP ratio (number of true positives divided by the number of false positives in the test set) of 1. Using such a threshold, the authors claim to predict 180 010 human interactions. We do not compare our predictions to this dataset because such a threshold of TP/FP > 1 does not correspond to a posterior odds threshold > 1. Depending on the positive-to-negative ratio used in the datasets, TP/FP > 1 might correspond to an average posterior odds ratio of 1. In contrast, the average posterior odds ratio of our LR400 dataset is above 700. In comparison, by using a threshold of TP/FP > 1 in our test set, we predict over 1 000 000 human interactions. We do not believe that the quality of this large number of predictions is high enough to warrant their publication since the great majority of these protein pairs achieve a posterior odds ratio below 1.

### Independent validation

**Figure 7:**
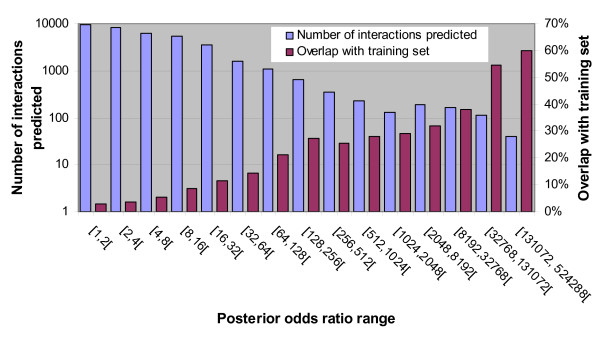
Overlap of different subsets of the LR400 dataset with the HPRD-derived training set. The number of interactions predicted and the proportion of overlap with the training set (which was derived from the HPRD) were calculated for subsets of the LR400 dataset of different posterior odds ratios.

Although the overlap between the LR400 dataset and the HPRD-derived positive training set is below 10% as shown in Figure 6C, the proportion of interactions common to these two sets is not equally distributed for all posterior odds ratios of interaction values. As shown in Figure 7, while less than 3% of the protein pairs predicted to interact at posterior odds ratios between 1 and 2 overlap with the HPRD dataset used for training, this value increases to over 50% for the highest scoring subsets of the LR400 dataset. These highest scoring predictions receive high likelihood ratios of interaction from all four predictive modules and represent the strongest examples of interaction as evaluated by our predictor. Such examples include interactions that allow the formation of well-known protein complexes such as the proteasome, the MCM protein complex involved in the initiation of genome replication, replication factor C, the TBP/TAF complex (TBP-associated factors) and the EIF complex (eukaryotic translation initiation factors). The highest scoring predictions in the LR400 dataset thus mainly represent interactions present in the HPRD dataset as well as interactions between proteins that have strong sequence identity to these known interacting pairs. However, as the posterior odds ratio decreases, the overlap between the predictions and the HPRD-derived training set decreases. Some subsets of quite high posterior odds have much smaller overlaps with the training set. For example, interactions predicted at posterior odds ratios between 128 and 2048 have a 20 to 30% overlap with the training set as shown in Figure 7. Although many of these novel predictions have not been previously investigated in the literature, there exists experimental evidence supporting a subset of these predictions which is not present in the June 2006 version of the HPRD used to train our predictor, thus providing independent validation of our method. Five such validated predictions are reported here:

-TCPTP was predicted to interact with STAT6 at a posterior odds ratio of 4300. It has been recently reported that TCPTP, the only protein tyrosine phosphatase known to localize to the nucleus, dephosphorylates STAT6 in this cellular compartment, which may in turn lead to the suppression of Interleukine-4 (IL-4) induced signaling [57].

-N-WASP and ARP3 achieve a predicted posterior odds ratio of interaction of 2700. A recent report suggested that the IQGAP1 protein can activate N-WASP thus changing its conformation and allowing it to bind the ARP2/3 complex, which in turn directs the generation of branched actin filaments required for the extension of a lamellipodium [58].

-The VAMP3-VTI1A interaction was predicted with a posterior odds ratio of 1518. Both these proteins are believed to be part of the SNARE (soluble *N*-ethylmaleimide-sensitive factor attachment protein receptor) family of proteins which are involved in membrane fusion events. VTI1A is a trans-Golgi-network-localized putative t-SNARE [59] and VAMP3 is an early/recycling endosomal v-SNARE [60]. These two proteins were recently shown to interact, leading to their functional implication in the post-Golgi retrograde transport step [61].

-CDK2 and MCM4 were predicted to interact at a posterior odds ratio of 62. CDK2 has recently been shown to phosphorylate MCM4, a subunit of a putative replicative helicase essential for DNA replication, on two distinct residues, leading to a change in its affinity to chromatin and its enrichment in the nucleolus [62].

-Sam68 and Smad2 achieve a predicted posterior odds ratio of 32. This interaction has been experimentally demonstrated by large-scale yeast-two-hybrid analysis of the Smad signaling system [63].

Our probabilistic predictor therefore not only reproduces and completes well-known protein complexes but also identifies novel interactions, a subset of which have been independently validated.

## Conclusion

The current human protein interaction map is estimated to be only 10% complete [18]. Here, we investigated the prediction of human protein-protein interactions in an effort to increase the coverage of the human interactome while simultaneously providing high quality predictions. By considering several different types of orthogonal and quite distinct features including expression, orthology, combined protein characteristics and local network topology, we predicted over 37000 human protein interactions and explored a subspace of the human interactome that has not been investigated by previous large interaction datasets. Our investigation led us to compare the influence of different training sets on the prediction accuracy. The use of randomly generated negative training examples and large negative-to-positive ratios in the training set generated the most accurate predictors in the context of our model. A comparison to other large human interaction datasets revealed the average false positive rate of our dataset to be 76%, which is much lower than the overall average for most large scale, currently available, human interaction datasets (experimental and computational) estimated to be 90% [18]. A subset of our novel predictions have been independently validated by identifying recent reports that experimentally investigated and confirmed that these protein pairs do interact. We provide all our predictions ranked according to the posterior odds ratio of interaction in Additional File 3. It is thus possible to restrict the dataset to the highest scoring protein pairs (and only choose for example, protein pairs that have an estimated true positive rate of interaction above 90%). By making this human interaction prediction dataset publicly available, it is our hope that it will help to identify the most high-confidence interactions, leading to a more complete and accurate human interaction map.

## Methods

### Datasets

In order to investigate the likelihood of interaction of human proteins, 62322 human protein sequences were downloaded from the International Protein Index (IPI) database (version 3.16) [64]. Some of these proteins are alternative transcripts of the same gene but can have distinct interaction partners. Known interactions were downloaded from the Human Protein Reference Database (HPRD; June 2006 version) [15]. Duplicate interactions and self-interactions were not considered. Additionally, some proteins were not recovered in the conversion between different identifiers. This resulted in 26896 distinct human protein interactions involving 7531 distinct human proteins present in the initial IPI dataset. The 26896 interactions from the June 2006 version of the HPRD were used as the positive dataset in the training/testing of the predictor. Two different sets of non-interacting protein pairs were investigated: the main analysis employed a randomly-generated negative dataset but this was also compared to a localization-derived negative dataset. Both non-interacting protein datasets were cleaned by removing all protein pairs that came from the positive dataset as well as protein pairs that were annotated as interacting in other databases (DIP [65]: 679 interactions, BIND [66]: 2650 interactions), or predicted to interact in other studies (OPHID [67]: 21815 interactions).

Of the 62322 human proteins from the initial IPI dataset, 22889 were characterized by at least one of the features that we considered to predict interaction (see the Features section). These 22889 human proteins are encoded by 16904 distinct genes and are referred to as the Informative Protein Set. The randomly-generated negative dataset used for the training and testing of the predictor was created by selecting protein pairs at random from the Informative Protein Set. In contrast, the localization-derived negative dataset was created by selecting protein pairs from the Informative Protein Set for which the HPRD [15] annotates one as being primarily in the plasma membrane and the other as primarily in the nucleus. Training and testing was performed with 5-fold cross-validation. In addition, positive to negative ratios of 1:1 and 1:100 were considered.

The predictions were compared to the literature-mined Ramani dataset [16], the orthology-derived Lehner prediction dataset [36] and the probabilistic Rhodes prediction dataset [46]. All three datasets identify the interactions by stating the names and/or gene locus IDs of the genes that encode the interacting proteins. In contrast, we work directly on the protein sequences and so related the gene annotations to our protein identifiers by extracting Entrez Gene IDs corresponding to the IPI protein entries from the IPI cross-reference files (for the IPI release 3.24) [64]. Ensembl gene identifiers (Ensembl 42) were also matched to Entrez Locus IDs (NCBI36) using BioMart [68].

Some gene-gene entries were not recovered in the conversion between different identifiers, or due to the deletion or replacement of some Entrez Locus IDs. Despite this, 37714 gene-gene interactions were recovered from the Rhodes dataset and 6132 interactions from the Ramani dataset as well as 64306 and 10454 interactions from the Lehner full and core datasets respectively.

### Learning method

Semi-naïve Bayes classifiers were used to measure the likelihood of interaction of two proteins given the presence of the features considered. This learning method was chosen because it allows the integration of highly heterogeneous data in a model that is easy to interpret and that can readily accommodate missing data. The transparency of the method allows the straightforward determination of which features are most predictive of interaction at the level of the whole proteome as well as for individual protein pairs.

The prediction of protein interaction is a binary problem which can be expressed in Bayesian formalism. We are interested in determining the posterior odds ratio of interaction of two proteins, given the presence of the features we are considering. This posterior odds ratio can be re-written using Bayes rule:

Opost=P(I|f1,..., fn)P(~I|f1,..., fn)=P(f1,..., fn|I)∗P(I)P(f1,..., fn)P(f1,..., fn|~I)∗P(~I)P(f1,..., fn)=P(f1,..., fn|I)∗P(I)P(f1,..., fn|~I)∗P(~I)=P(I)P(~I)∗P(f1,..., fn|I)P(f1,..., fn|~I)=Oprior∗LR(f1,...,fn)
 MathType@MTEF@5@5@+=feaafiart1ev1aaatCvAUfKttLearuWrP9MDH5MBPbIqV92AaeXatLxBI9gBaebbnrfifHhDYfgasaacH8akY=wiFfYdH8Gipec8Eeeu0xXdbba9frFj0=OqFfea0dXdd9vqai=hGuQ8kuc9pgc9s8qqaq=dirpe0xb9q8qiLsFr0=vr0=vr0dc8meaabaqaciaacaGaaeqabaqabeGadaaakeaafaqadeqbbaaaaeaacqqGpbWtdaWgaaWcbaGaeeiCaaNaee4Ba8Maee4CamNaeeiDaqhabeaakiabg2da9maalaaabaGaeeiuaa1aaeWaaeaacqqGjbqscqGG8baFcqqGMbGzdaWgaaWcbaGaeeymaedabeaakiabbYcaSiabb6caUiabb6caUiabb6caUiabbYcaSiabbccaGiabbAgaMnaaBaaaleaacqqGUbGBaeqaaaGccaGLOaGaayzkaaaabaGaeeiuaa1aaeWaaeaacqGG+bGFcqqGjbqscqGG8baFcqqGMbGzdaWgaaWcbaGaeeymaedabeaakiabbYcaSiabb6caUiabb6caUiabb6caUiabbYcaSiabbccaGiabbAgaMnaaBaaaleaacqqGUbGBaeqaaaGccaGLOaGaayzkaaaaaaqaaiabg2da9maalaaabaWaaSaaaeaacqqGqbaudaqadaqaaiabbAgaMnaaBaaaleaacqqGXaqmaeqaaOGaeeilaWIaeeOla4IaeeOla4IaeeOla4IaeeilaWIaeeiiaaIaeeOzay2aaSbaaSqaaiabb6gaUbqabaGccqGG8baFcqqGjbqsaiaawIcacaGLPaaacqGHxiIkcqqGqbaudaqadaqaaiabbMeajbGaayjkaiaawMcaaaqaaiabbcfaqnaabmaabaGaeeOzay2aaSbaaSqaaiabbgdaXaqabaGccqqGSaalcqqGUaGlcqqGUaGlcqqGUaGlcqqGSaalcqqGGaaicqqGMbGzdaWgaaWcbaGaeeOBa4gabeaaaOGaayjkaiaawMcaaaaaaeaadaWcaaqaaiabbcfaqnaabmaabaGaeeOzay2aaSbaaSqaaiabbgdaXaqabaGccqqGSaalcqqGUaGlcqqGUaGlcqqGUaGlcqqGSaalcqqGGaaicqqGMbGzdaWgaaWcbaGaeeOBa4gabeaakiabcYha8jabc6ha+jabbMeajbGaayjkaiaawMcaaiabgEHiQiabbcfaqnaabmaabaGaeiOFa4NaeeysaKeacaGLOaGaayzkaaaabaGaeeiuaa1aaeWaaeaacqqGMbGzdaWgaaWcbaGaeeymaedabeaakiabbYcaSiabb6caUiabb6caUiabb6caUiabbYcaSiabbccaGiabbAgaMnaaBaaaleaacqqGUbGBaeqaaaGccaGLOaGaayzkaaaaaaaaaeaacqGH9aqpdaWcaaqaaiabbcfaqnaabmaabaGaeeOzay2aaSbaaSqaaiabbgdaXaqabaGccqqGSaalcqqGUaGlcqqGUaGlcqqGUaGlcqqGSaalcqqGGaaicqqGMbGzdaWgaaWcbaGaeeOBa4gabeaakiabcYha8jabbMeajbGaayjkaiaawMcaaiabgEHiQiabbcfaqnaabmaabaGaeeysaKeacaGLOaGaayzkaaaabaGaeeiuaa1aaeWaaeaacqqGMbGzdaWgaaWcbaGaeeymaedabeaakiabbYcaSiabb6caUiabb6caUiabb6caUiabbYcaSiabbccaGiabbAgaMnaaBaaaleaacqqGUbGBaeqaaOGaeiiFaWNaeiOFa4NaeeysaKeacaGLOaGaayzkaaGaey4fIOIaeeiuaa1aaeWaaeaacqGG+bGFcqqGjbqsaiaawIcacaGLPaaaaaaabaGaeyypa0ZaaSaaaeaacqqGqbaudaqadaqaaiabbMeajbGaayjkaiaawMcaaaqaaiabbcfaqnaabmaabaGaeiOFa4NaeeysaKeacaGLOaGaayzkaaaaaiabgEHiQmaalaaabaGaeeiuaa1aaeWaaeaacqqGMbGzdaWgaaWcbaGaeeymaedabeaakiabbYcaSiabb6caUiabb6caUiabb6caUiabbYcaSiabbccaGiabbAgaMnaaBaaaleaacqqGUbGBaeqaaOGaeiiFaWNaeeysaKeacaGLOaGaayzkaaaabaGaeeiuaa1aaeWaaeaacqqGMbGzdaWgaaWcbaGaeeymaedabeaakiabbYcaSiabb6caUiabb6caUiabb6caUiabbYcaSiabbccaGiabbAgaMnaaBaaaleaacqqGUbGBaeqaaOGaeiiFaWNaeiOFa4NaeeysaKeacaGLOaGaayzkaaaaaaqaaiabg2da9iabb+eapnaaBaaaleaacqqGWbaCcqqGYbGCcqqGPbqAcqqGVbWBcqqGYbGCaeqaaOGaey4fIOIaeeitaWKaeeOuaiLaeiikaGIaeeOzay2aaSbaaSqaaiabigdaXaqabaGccqGGSaalcqGGUaGlcqGGUaGlcqGGUaGlcqGGSaalcqqGMbGzdaWgaaWcbaGaeeOBa4gabeaakiabcMcaPaaaaaa@0F81@

where I is a binary variable representing interaction, ~I represents non-interaction, f_1 _through f_n _are the features we are considering, O_prior _is the prior odds ratio and LR is the likelihood ratio.

If the features considered are independent, the likelihood ratio LR can be calculated as the product of the individual likelihood ratios with respect to the features considered separately. If the features are not independent, all possible combinations of all states of these features must be considered, which can be computationally quite intensive. In the independent case, the likelihood ratio can be calculated as:

LR(f1,...,fn)=[P(f1,..., fn|I)P(f1,..., fn|~I)]=∏i=1n[P(fi|I)P(fi|~I)]
 MathType@MTEF@5@5@+=feaafiart1ev1aaatCvAUfKttLearuWrP9MDH5MBPbIqV92AaeXatLxBI9gBaebbnrfifHhDYfgasaacH8akY=wiFfYdH8Gipec8Eeeu0xXdbba9frFj0=OqFfea0dXdd9vqai=hGuQ8kuc9pgc9s8qqaq=dirpe0xb9q8qiLsFr0=vr0=vr0dc8meaabaqaciaacaGaaeqabaqabeGadaaakeaafaqadeGabaaabaGaeeitaWKaeeOuaiLaeiikaGIaeeOzay2aaSbaaSqaaiabigdaXaqabaGccqGGSaalcqGGUaGlcqGGUaGlcqGGUaGlcqGGSaalcqqGMbGzdaWgaaWcbaGaeeOBa4gabeaakiabcMcaPiabg2da9maadmaabaWaaSaaaeaacqqGqbaudaqadaqaaiabbAgaMnaaBaaaleaacqqGXaqmaeqaaOGaeeilaWIaeeOla4IaeeOla4IaeeOla4IaeeilaWIaeeiiaaIaeeOzay2aaSbaaSqaaiabb6gaUbqabaGccqGG8baFcqqGjbqsaiaawIcacaGLPaaaaeaacqqGqbaudaqadaqaaiabbAgaMnaaBaaaleaacqqGXaqmaeqaaOGaeeilaWIaeeOla4IaeeOla4IaeeOla4IaeeilaWIaeeiiaaIaeeOzay2aaSbaaSqaaiabb6gaUbqabaGccqGG8baFcqGG+bGFcqqGjbqsaiaawIcacaGLPaaaaaaacaGLBbGaayzxaaaabaGaeyypa0ZaaebCaeaadaWadaqaamaalaaabaGaeeiuaa1aaeWaaeaacqqGMbGzdaWgaaWcbaGaeeyAaKgabeaakiabcYha8jabbMeajbGaayjkaiaawMcaaaqaaiabbcfaqnaabmaabaGaeeOzay2aaSbaaSqaaiabbMgaPbqabaGccqGG8baFcqGG+bGFcqqGjbqsaiaawIcacaGLPaaaaaaacaGLBbGaayzxaaaaleaacqqGPbqAcqGH9aqpcqaIXaqmaeaacqqGUbGBa0Gaey4dIunaaaaaaa@7A83@

The likelihood ratios for the different features considered can be estimated by evaluating the ratio of the proportion of interacting and non-interacting proteins for which a particular state of the feature is true in the training set (i.e. by determining to which bin of the feature the protein pair belongs, for every protein pair in the positive and negative training sets). More precisely, the training step consisted of calculating the respective proportions of positive and negative examples that fall into each bin of the feature(s) considered (i.e. that have a particular state). The likelihood ratio of interaction for a given state is simply the ratio of the proportion of all positives that have that state divided by the proportion of all negatives that have that same state. When a particular state of a feature occurs only in positive examples (known interacting proteins), the likelihoods are set to the highest non-infinite value of any state for that feature (to avoid infinite values). Additionally, when no data are available for a specific feature (for a given pair of proteins), the likelihood of the feature is set to 1.0. For a detailed calculation of the likelihoods see Additional File 4.

### Prior odds ratio estimate

The prior odds ratio (O_prior_) is difficult to estimate because we do not know all the true interactions, even for a small subset of proteins. The prior odds ratio of interaction for yeast was estimated by combining all protein-protein interactions (but only those related to direct physical interactions, and no entries derived by synthetic lethal-type experiments) from the BIND, DIP and GRID databases [65,66,69]. This subset of interactions contains 36466 distinct interactions involving 5202 distinct proteins, thus resulting in a prior odds ratio of 1/370. This is most likely a conservative estimate since a certain proportion of interactions remain unknown and so when more data become available, the prior odds ratio will increase. For human proteins, 12191 distinct interactions were recovered, involving 5164 human proteins from the September 2005 version of the HPRD [15] and 26896 distinct interactions involving 7531 human proteins from the June 2006 version, leading respectively to prior odds estimates of 1/1093 and 1/1053. However, taking the subset of 5164 proteins from the September 2005 version that are seen in the June 2006 version (20842 distinct interactions), gave a prior odds of interaction estimate of 1/639. Thus, between the two releases of the HPRD, there was a large increase in the number of interactions for this subset of proteins and this is likely to continue for at least the next few releases. Accordingly, it is reasonable to conclude that there are not enough known human interactions to calculate a realistic and stable estimate of the prior odds ratio of interactions for human. As a consequence, a prior odds ratio of 1/400 was used for all work in the paper, which is similar to the estimate for yeast and is likely still an underestimate of the true value.

### Features

Seven distinct features combined into five modules were investigated as summarized in Table 1 and described below.

#### 1. Expression module

Expression data were downloaded from the Gene Expression Omnibus [70]. The GDS596 dataset was used which examines gene expression profiles from 79 physiologically normal tissues obtained from various sources [71]. Expression data were recovered for 10642 distinct transcripts in 158 different arrays (2 arrays per tissue). Pearson correlations were calculated for all 56620761 transcript pairs and correlation values were grouped into 20 bins of increasing co-expression.

#### 2. Orthology module

Orthology maps between human and yeast, worm and fly were downloaded from the InParanoid database [72]. Interaction datasets for model organisms were downloaded from the BIND [66], DIP [65] and GRID [69] databases. Orthology interaction data were classified into 13 bins. High, medium and low confidence bins were defined for human protein pairs that have interacting orthologs in either yeast, fly or worm (for a total of 9 bins). The high confidence bins were populated by human protein pairs that have interacting orthologs that both achieve an InParanoid score of 1 (i.e. both proteins involved in an interaction in another organism are respectively the best orthology match for the two human proteins under consideration). The medium confidence bins were populated by human protein pairs that have interacting orthologs but only one of the interacting orthologs has an InParanoid score of 1. The low confidence bins were filled by human protein pairs that have interacting orthologs according to InParanoid but neither achieves a score of 1 (i.e. neither is the best match for the two human proteins under consideration). The orthology module has four additional bins: two bin for human pairs that have interacting paralogs in human (a medium and a low confidence bin which use the same definition as above for the model organisms), one bin for human pairs that have interacting homologs in more than one organism (these can be orthologs in yeast, worm or fly, or paralogs in human) and one bin for human pairs that have only non-interacting orthologs.

#### 3. Combined module

This module incorporates three distinct features in a non-naïve Bayesian framework: subcellular localization, domain co-occurrence and post-translational modification co-occurrence.

##### Subcellular localization

PSLT (Protein Subcellular Localization Tool) subcellular localization predictions [54] were used to classify protein pairs in one of four groups: pairs of proteins predicted to be in the same compartment, pairs of proteins predicted to be in neighboring compartments (cytosol-nucleus, endoplasmic reticulum-Golgi, Golgi-cytosol, cytosol-plasma membrane, and plasma membrane-secreted), pairs of proteins predicted in different non-neighboring compartments and pairs of proteins for which there were no localization predictions. Neighboring compartments were chosen as compartment pairs sharing a high proportion of proteins, as investigated previously [54].

##### Co-occurrence of domains

The chi-square test was used as a measure of the likelihood of co-occurrence of specific InterPro domains and motifs [73] in protein pairs. Chi-square scores were calculated for all pairs of domains/motifs that occurred in the training data and were then grouped into 5 bins of increasing value. Additionally, Pfam [74] domain pairs known to interact from three-dimensional structures [75] were included in the highest Chi-square score bin. When protein pairs contained more than one domain pair, the domain pair assigned to the highest Chi-square score bin was used to assign a likelihood of interaction.

##### Post-translational modification (PTM) pair co-occurrence

Likelihoods were assessed using a PTM pair enrichment score calculated as the probability of co-occurrence of two specific PTMs in all pairs of interacting protein pairs divided by the probability of occurrence of both of these PTMs separately:

PTM_score=P(PTM[i],PTM[j]|I)P(PTM[i]|I)∗P(PTM[j]|I)
 MathType@MTEF@5@5@+=feaafiart1ev1aaatCvAUfKttLearuWrP9MDH5MBPbIqV92AaeXatLxBI9gBaebbnrfifHhDYfgasaacH8akY=wiFfYdH8Gipec8Eeeu0xXdbba9frFj0=OqFfea0dXdd9vqai=hGuQ8kuc9pgc9s8qqaq=dirpe0xb9q8qiLsFr0=vr0=vr0dc8meaabaqaciaacaGaaeqabaqabeGadaaakeaacqqGqbaucqqGubavcqqGnbqtcqqGFbWxcqqGZbWCcqqGJbWycqqGVbWBcqqGYbGCcqqGLbqzcqGH9aqpdaWcaaqaaiabbcfaqnaabmaabaGaeeiuaaLaeeivaqLaeeyta00aamWaaeaacqqGPbqAaiaawUfacaGLDbaacqqGSaalcqqGqbaucqqGubavcqqGnbqtdaWadaqaaiabbQgaQbGaay5waiaaw2faaiabbYha8jabbMeajbGaayjkaiaawMcaaaqaaiabbcfaqnaabmaabaGaeeiuaaLaeeivaqLaeeyta00aamWaaeaacqqGPbqAaiaawUfacaGLDbaacqqG8baFcqqGjbqsaiaawIcacaGLPaaacqGHxiIkcqqGqbaudaqadaqaaiabbcfaqjabbsfaujabb2eannaadmaabaGaeeOAaOgacaGLBbGaayzxaaGaeeiFaWNaeeysaKeacaGLOaGaayzkaaaaaaaa@660B@

where PTM[i] and PTM[j] are distinct PTMs and I is the set of all interacting proteins that were used to train the predictor. The annotations of PTMs in human proteins were downloaded from UniProt [76] and HPRD [15]. PTM instances described as "predicted", "probable" or "possible" were excluded, leaving 3439 distinct proteins with PTM annotations in the training set. The PTM pair enrichment scores were grouped into 4 bins of increasing value.

The localization, co-occurrence of domains, and PTMs were considered simultaneously to measure their predictive power in assessing the likelihood of protein interaction. To do this, all possible combinations of the 4 localization bins, 5 chi-square domain-co-occurrence bins and 4 PTM_score bins were investigated and are referred to as the **combined module**.

#### 4. Disorder module

It has been suggested that unstructured regions of proteins are often involved in binding interactions, particularly in the case of transient interactions [77]. Protein intrinsic disorder was predicted for all proteins considered by using the VSL2B predictor [78]. The disorder score for protein pairs was then calculated as the sum of percent disorder for each protein of the pair. Disorder scores were grouped into 6 bins of increasing value.

The Expression, Orthology, Combined and Disorder modules are referred collectively as the **Group A modules**. Likelihood ratios for each of the Group A modules are illustrated in Figure 1A (see Additional File 4 for complete likelihood ratios for every possible state of these modules and for detailed calculations of these likelihood ratios).

#### 5. Transitive module

**Figure 2:**
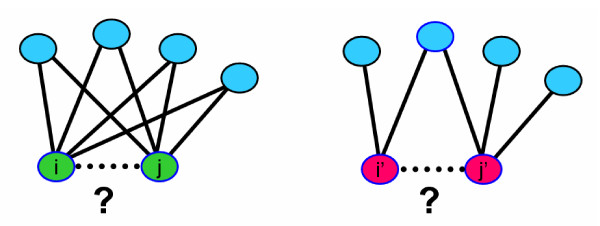
Transitive module hypothesis. The Transitive module investigates whether two proteins (such as i and j) that share many common interactors and have few additional interactors that are not common to both proteins are more likely to interact than two proteins (such as i' and j') that share few common interactors.

The transitive module works on the premise that a pair of proteins is more likely to interact if it shares interacting partners. It does this by considering the local topology of the network predicted by the integration of the Group A modules as depicted in Figure 2. Thus, the transitive module takes as input the product of the likelihood ratios of all other modules considered by the predictor (as illustrated in Figure 1B). For each pair of proteins in the training set, the product of the likelihood ratios from all other modules (referred to as the preliminary score (PS) in Figure 1) was calculated for all protein pairs neighboring the pair (i.e. all protein pairs which involve one protein from the initial protein pair under study and for which it is possible to calculate such a score). All preliminary scores above 10 were kept. This parameter was determined empirically. A neighborhood topology score T was then calculated as follows:

T=∑e∈Ecse1+|Ei\Ec|+|Ej\Ec|
 MathType@MTEF@5@5@+=feaafiart1ev1aaatCvAUfKttLearuWrP9MDH5MBPbIqV92AaeXatLxBI9gBaebbnrfifHhDYfgasaacH8akY=wiFfYdH8Gipec8Eeeu0xXdbba9frFj0=OqFfea0dXdd9vqai=hGuQ8kuc9pgc9s8qqaq=dirpe0xb9q8qiLsFr0=vr0=vr0dc8meaabaqaciaacaGaaeqabaqabeGadaaakeaacqqGubavcqGH9aqpdaWcaaqaamaaqafabaGaee4Cam3aaSbaaSqaaiabbwgaLbqabaaabaGaeeyzauMaeyicI4Saeeyrau0aaSbaaWqaaiabbogaJbqabaaaleqaniabggHiLdaakeaacqaIXaqmcqGHRaWkcqGG8baFcqqGfbqrdaWgaaWcbaGaeeyAaKgabeaakiabcYfaCjabbweafnaaBaaaleaacqqGJbWyaeqaaOGaeiiFaWNaey4kaSIaeiiFaWNaeeyrau0aaSbaaSqaaiabbQgaQbqabaGccqGGCbaxcqqGfbqrdaWgaaWcbaGaee4yamgabeaakiabcYha8baaaaa@4F06@

where E_c _is the set of edges that connect proteins i and j to their common interactors, E_i _is the set of edges that involve protein i, s_e _is the score (likelihood ratio) of edge e and E_i_\E_c _refers to the set difference of E_i _and E_c_. For a given set of neighbors, T increases as the interactions with these neighbors become more likely (as the sum of s_e _increases). Additionally, the topology score T of a pair of proteins increases as the proportion of likely interactors that these two proteins share increases. The topology scores were grouped into 5 bins of increasing value. It should be noted that the neighborhood topology score calculated for a given protein pair does not consider the preliminary score assigned to that protein pair. It only considers the preliminary scores of its neighbors and so is truly based on the local network topology around that protein pair. Accordingly, the likelihood ratio the transitive module outputs for a given protein pair is independent of the likelihood ratio calculated by the Group A modules for this same protein pair.

### Correlation analysis

The Pearson correlation between pairs of modules was estimated by taking 150 samples of 10000 protein pairs each and calculating the Pearson correlation of the likelihood ratios for the two modules considered, for each sample. The reported correlation values are the average of the 150 experiments. Samples of the protein pair space were taken instead of considering the whole space as this was more computationally tractable.

### Accuracy measurements

The accuracy of the predictors was measured by performing five-fold cross validation experiments in which the datasets were randomly divided into five non-overlapping sets, four of which were used to train the predictor while the fifth was used to test the prediction accuracy. The accuracy reported is the average measured for all combinations of training and testing sets using these five sets. Testing was done by predicting the total likelihood scores for all protein pairs in the test set using the models computed in the training phase and then counting the number of pairs that were well predicted. We used the area under partial ROC curves as a measure of accuracy. Receiver operator characteristic (ROC) curves plot the true positive rate versus the false positive rate over their full range of possible values. In some circumstances, it is more informative to use partial ROC curves (ROCn curves) which illustrate the number of true positives identified by the predictor that score higher than the n highest scoring negatives, plotted for all values from 0 to n. There are many more negatives than positives in our datasets and this is also thought to be true for the full protein interaction networks we are modeling. Since the aim is to identify the largest number of true interacting pairs while leaving out as many non-interacting pairs as possible, it is most informative to measure the performance of the predictor under conditions of very low false-positive rates. Accordingly, ROC50 and ROC100 curves were analyzed because given the size of the datasets, these curves consider all the protein pairs predicted to have a posterior odds ratio above 1.0, for all the predictors investigated. The area under ROC curves is often used as a summary measure of accuracy. For ROCn curves, it can be calculated as

AUCROCn=1nT∗(∑i=1nTi)
 MathType@MTEF@5@5@+=feaafiart1ev1aaatCvAUfKttLearuWrP9MDH5MBPbIqV92AaeXatLxBI9gBaebbnrfifHhDYfgasaacH8akY=wiFfYdH8Gipec8Eeeu0xXdbba9frFj0=OqFfea0dXdd9vqai=hGuQ8kuc9pgc9s8qqaq=dirpe0xb9q8qiLsFr0=vr0=vr0dc8meaabaqaciaacaGaaeqabaqabeGadaaakeaacqqGbbqqcqqGvbqvcqqGdbWqdaWgaaWcbaGaeeOuaiLaee4ta8Kaee4qamKaeeOBa4gabeaakiabg2da9maalaaabaGaeGymaedabaGaeeOBa4MaeeivaqfaaiabgEHiQiabcIcaOmaaqahabaGaeeivaq1aaSbaaSqaaiabbMgaPbqabaaabaGaeeyAaKMaeyypa0JaeGymaedabaGaeeOBa4ganiabggHiLdGccqGGPaqkaaa@45C9@

where i takes on values from 1 to n, T is the total number of positives in the test set and T_i _is the number of positives that score higher than the i^th ^highest scoring negative.

## Authors' contributions

MSS conceived and designed the study, created and implemented the predictor, analyzed the predictions and drafted the manuscript. GJB participated in the design of the study, the analysis of the predictions and the writing of the manuscript. Both authors read and approved the final manuscript.

## Supplementary Material

Additional File 1 High scoring InterPro domain pairs. List of InterPro domain pairs that achieve highest chi-square score of co-occurrence in our set of positive interactors. Click here for file

Additional File 2 High scoring post-translational modification pairs. List of high-scoring post-translational modification pairs. Click here for file

Additional File 3 All LR400 predicted interactions ranked. All human protein pairs predicted to have a likelihood ratio of interaction greater than 400 and thus a posterior odds ratio of interaction greater than 1. Click here for file

Additional file 4 Additional methods. In depth description of the calculation of likelihood ratios for the modules. Click here for file
