# Supplementary material for: Probabilistic prediction and ranking of human protein-protein interactions
Source: BMC Bioinformatics. 2007 Jul 5;8:239. doi: 10.1186/1471-2105-8-239 (PMC1939716; doi:10.1186/1471-2105-8-239)
Supplement: Additional file 4 — Additional methods. In depth description of the calculation of likelihood ratios for the modules. [file 1471-2105-8-239-S4.pdf]

ADDITIONAL MATERIALS AND METHODS

Learning method

The prediction of protein interaction is a binary problem which can be expressed using Bayesian formalism. We are interested in determining the posterior odds ratio of interaction of two proteins, given the presence of the features we are considering. This posterior odds ratio can be re-written using Bayes rule:

$$
\begin{aligned}
O_{post} &= \frac{P(I \mid f_1,\ldots,f_n)}{P(\sim I \mid f_1,\ldots,f_n)} \\
&= \frac{\dfrac{P(f_1,\ldots,f_n \mid I) * P(I)}{P(f_1,\ldots,f_n)}}{\dfrac{P(f_1,\ldots,f_n \mid \sim I) * P(\sim I)}{P(f_1,\ldots,f_n)}} \\
&= \frac{P(f_1,\ldots,f_n \mid I) * P(I)}{P(f_1,\ldots,f_n \mid \sim I) * P(\sim I)} \\
&= \frac{P(I)}{P(\sim I)} * \frac{P(f_1,\ldots,f_n \mid I)}{P(f_1,\ldots,f_n \mid \sim I)} \\
&= O_{prior} \quad * \quad LR(f_1, \ldots, f_n)
\end{aligned}
$$

where I is a binary variable representing interaction (and ~I represents non-interaction) and f_1 through f_n are the features we are considering.

If the features considered are independent, the likelihood ratio LR can be calculated as the product of the individual likelihood ratios with respect to the features considered separately. If the features are not independent, all possible combinations of all states of these features must be considered,

which can be computationally quite intensive. In the independent case, the likelihood ratio can be calculated as:

$$\mathrm{LR}(f_1, \ldots, f_n) = \left[\frac{P(f_1,\ldots,f_n \mid I)}{P(f_1,\ldots,f_n \mid \sim I)}\right]$$

$$= \prod_{i=1}^{n}\left[\frac{P(f_i \mid I)}{P(f_i \mid \sim I)}\right]$$

The likelihoods for the different features considered were estimated by evaluating the ratio of the proportion of interacting and non-interacting proteins for which a particular state of the feature is true in the training set (ie by determining to which bin of the feature state the protein pair belongs, for every protein pair in the positive and negative training sets). A detailed calculation of the likelihood ratios is shown below, for each module.

Expression module likelihood ratios:

Bins	Number of positives	Number of negatives	P(bin|pos)	P(bin|neg)	Likelihood ratio
[-0.6, -0.5]	3	31	0.000203	5.71E-05	3.560943
]-0.5, -0.4]	9	700	0.00061	0.001289	0.473097
]-0.4, -0.3]	75	5216	0.005081	0.009604	0.529089
]-0.3, -0.2]	427	23546	0.02893	0.043354	0.667292
]-0.2, -0.1]	1161	72056	0.078659	0.132672	0.592881
]-0.1, 0.0]	2298	128389	0.155691	0.236394	0.658609
]0.0, 0.1]	2165	106374	0.14668	0.195859	0.748907
]0.1, 0.2]	1829	72746	0.123916	0.133942	0.925145
]0.2, 0.3]	1516	48150	0.10271	0.088655	1.158533
]0.3, 0.4]	1387	33386	0.09397	0.061471	1.528683
]0.4, 0.5]	1185	23350	0.080285	0.042993	1.867398
]0.5, 0.6]	1057	15379	0.071612	0.028316	2.52902
]0.6, 0.7]	765	8992	0.051829	0.016556	3.130477
]0.7, 0.8]	553	3930	0.037466	0.007236	5.177714
]0.8, 0.9]	227	756	0.015379	0.001392	11.04866
]0.9, 1.0]	103	114	0.006978	0.00021	33.24588
**Total**	14760	543115	1	1	

Orthology module likelihood ratios:

Bins	Number of positives	Number of negatives	P(bin|pos)	P(bin|neg)	Likelihood ratio
**Yeast high**	376	91	0.037532	0.000158	237.5156
**Yeast medium**	161	194	0.016071	0.000337	47.70565
**Yeast low**	48	108	0.004791	0.000188	25.54837
**Fly high**	16	13	0.001597	2.26E-05	70.74933
**Fly medium**	13	16	0.001298	2.78E-05	46.70561
**Fly low**	5	10	0.000499	1.74E-05	28.74191
**Worm high**	26	9	0.002595	1.56E-05	166.0644
**Worm medium**	32	24	0.003194	4.17E-05	76.64511
**Worm low**	19	8	0.001897	1.39E-05	136.5241
**Human medium**	36	2	0.003594	3.47E-06	1034.709
**Human low**	210	28	0.020962	4.86E-05	431.1287
**More than one organism**	105	5	0.010481	8.68E-06	1207.16
**Only non-interacting orthologues**	8971	575365	0.895488	0.999118	0.896279
**Total**	10018	575873	1	1	

Combined module likelihood ratios:

Bins			Number of positives	Number of negatives	P(bin|pos)	P(bin|neg)	Likelihood ratio
Dom	Loc	PTM					
0	0	1	63	6377	0.003003	0.007938	0.378
0	0	2	839	16730	0.039996	0.020825	1.921
0	0	3	17	57	0.00081	7.1E-05	11.422
0	1	0	1088	308368	0.051866	0.383851	0.135
0	1	1	49	2577	0.002336	0.003208	0.728
0	1	2	378	9586	0.01802	0.011932	1.510
0	1	3	12	49	0.000572	6.1E-05	9.379
0	2	0	1510	175157	0.071984	0.218032	0.330
0	2	1	45	1287	0.002145	0.001602	1.339
0	2	2	815	5457	0.038852	0.006793	5.720
0	2	3	8	23	0.000381	2.86E-05	13.32
0	3	0	2764	177265	0.131763	0.220656	0.597
0	3	1	51	988	0.002431	0.00123	1.977
0	3	2	1638	5745	0.078086	0.007151	10.919
0	3	3	46	29	0.002193	3.61E-05	60.747
1	0	0	44	6373	0.002098	0.007933	0.264
1	0	1	1	267	4.77E-05	0.000332	0.143
1	0	2	18	177	0.000858	0.00022	3.895
1	0	3	0	1	0	1.24E-06	0
1	1	0	37	1557	0.001764	0.001938	0.910
1	1	1	2	20	9.53E-05	2.49E-05	3.830
1	1	2	17	104	0.00081	0.000129	6.260
1	1	3	0	0	0	0	-
1	2	0	74	1651	0.003528	0.002055	1.717
1	2	1	1	37	4.77E-05	4.61E-05	1.035
1	2	2	61	146	0.002908	0.000182	16.001
1	2	3	3	0	0.000143	0	612.75
1	3	0	123	2186	0.005864	0.002721	2.155
1	3	1	5	34	0.000238	4.23E-05	5.632
1	3	2	121	218	0.005768	0.000271	21.257
1	3	3	2	0	9.53E-05	0	612.75
2	0	0	153	24173	0.007294	0.03009	0.242
2	0	1	1	253	4.77E-05	0.000315	0.151
2	0	2	147	640	0.007008	0.000797	8.796
2	0	3	0	1	0	1.24E-06	0
2	1	0	172	3805	0.008199	0.004736	1.731
2	1	1	5	61	0.000238	7.59E-05	3.139
2	1	2	111	315	0.005292	0.000392	13.495
2	1	3	2	2	9.53E-05	2.49E-06	38.297
2	2	0	316	5980	0.015064	0.007444	2.0237
2	2	1	12	100	0.000572	0.000124	4.596
2	2	2	301	577	0.014349	0.000718	19.978
2	2	3	10	11	0.000477	1.37E-05	34.815

**2**	**3**	**0**	760	8300	0.03623	0.010332	3.507
**2**	**3**	**1**	14	81	0.000667	0.000101	6.619
**2**	**3**	**2**	711	808	0.033894	0.001006	33.699
**2**	**3**	**3**	22	10	0.001049	1.24E-05	84.253
**3**	**0**	**0**	624	18773	0.029747	0.023368	1.273
**3**	**0**	**1**	10	276	0.000477	0.000344	1.388
**3**	**0**	**2**	303	574	0.014444	0.000715	20.216
**3**	**0**	**3**	4	14	0.000191	1.74E-05	10.942
**3**	**1**	**0**	289	2348	0.013777	0.002923	4.714
**3**	**1**	**1**	7	45	0.000334	5.6E-05	5.957
**3**	**1**	**2**	150	208	0.007151	0.000259	27.618
**3**	**1**	**3**	18	3	0.000858	3.73E-06	229.781
**3**	**2**	**0**	883	3984	0.042094	0.004959	8.488
**3**	**2**	**1**	34	72	0.001621	8.96E-05	18.085
**3**	**2**	**2**	571	498	0.02722	0.00062	43.911
**3**	**2**	**3**	10	10	0.000477	1.24E-05	38.297
**3**	**3**	**0**	1930	6863	0.092006	0.008543	10.770
**3**	**3**	**1**	49	84	0.002336	0.000105	22.340
**3**	**3**	**2**	1326	764	0.063212	0.000951	66.468
**3**	**3**	**3**	49	27	0.002336	3.36E-05	69.502
**4**	**0**	**0**	208	687	0.009916	0.000855	11.595
**4**	**0**	**1**	3	0	0.000143	0	612.75
**4**	**0**	**2**	58	9	0.002765	1.12E-05	246.802
**4**	**0**	**3**	0	0	0	0	-
**4**	**1**	**0**	50	34	0.002384	4.23E-05	56.319
**4**	**1**	**1**	2	0	9.53E-05	0	612.75
**4**	**1**	**2**	56	10	0.00267	1.24E-05	214.463
**4**	**1**	**3**	6	0	0.000286	0	612.75
**4**	**2**	**0**	442	194	0.021071	0.000241	87.254
**4**	**2**	**1**	17	3	0.00081	3.73E-06	217.016
**4**	**2**	**2**	317	31	0.015112	3.86E-05	391.617
**4**	**2**	**3**	0	0	0	0	-
**4**	**3**	**0**	651	235	0.031034	0.000293	106.091
**4**	**3**	**1**	22	2	0.001049	2.49E-06	421.266
**4**	**3**	**2**	287	21	0.013682	2.61E-05	523.39
**4**	**3**	**3**	32	2	0.001525	2.49E-06	612.75
**Total**			20977	803354	1	1	

The domain, ptm and localization bins are ranked from 0 to 4 where the 0 bin represents the lowest scoring bin and 4 represents the highest scoring bin.

Transitive module likelihood ratios:

Bins (Topology score range)	Number of positives	Number of negatives	P(bin|pos)	P(bin|neg)	Likelihood ratio
**[0,25]**	15319	342318	0.864406	0.998923	0.865338
**]25, 100]**	730	155	0.041192	0.000452	91.07015
**]100, 400]**	593	96	0.033461	0.00028	119.45
**]400, 1600]**	488	68	0.027536	0.000198	138.7701
**> 1600**	592	50	0.033405	0.000146	228.9479
**Total**	17722	342687	1	1	

When a particular state of a feature occurs only in positive examples (known interacting proteins), the likelihoods are set to the highest non-infinite value of any state for that feature (to avoid infinite values). Additionally, when no data are available for a specific feature (for a given pair of proteins), the likelihood of the feature is set to 1.0.
